# Physiologic Response Evaluation of Human Foetal Osteoblast Cells within Engineered 3D-Printed Polylactic Acid Scaffolds

**DOI:** 10.3390/biology12030424

**Published:** 2023-03-10

**Authors:** Maria Giovanna Rizzo, Nicoletta Palermo, Paola Alibrandi, Emanuele Luigi Sciuto, Costantino Del Gaudio, Vincenzo Filardi, Barbara Fazio, Antonella Caccamo, Salvatore Oddo, Giovanna Calabrese, Sabrina Conoci

**Affiliations:** 1 Department of Chemical, Biological, Pharmaceutical and Environmental Sciences, University of Messina, Viale Ferdinando Stagno d’Alcontres, 31, 98168 Messina, Italy; 2 Italian Space Agency, Via del Politecnico snc, 00133 Rome, Italy; 3 Department TTO, Piazza Pugliatti 1, 98122 Messina, Italy; 4 CNR URT Lab SENS, Beyond NANO, Viale Ferdinando Stagno d’Alcontres 31, 98166 Messina, Italy; 5 CNR-IPCF, Istituto per i Processi Chimico-Fisici, Viale F. Stagno D’Alcontres 37, 98158 Messina, Italy; 6 Department of Chemistry “Giacomo Ciamician”, University of Bologna, 40126 Bologna, Italy

**Keywords:** bone tissue engineering, polylactic acid scaffolds, 3D-printing, human foetal osteoblast cells, osteoconductivity, osteoinductivity

## Abstract

**Simple Summary:**

Large bone defect treatments have always represented an important challenge in clinical practice and created a large demand for more efficacious regenerative approaches. The bone tissue engineering approach offered a new alternative to conventional bone grafts, addressing all clinical needs. Among the most used biomaterials for bone tissue engineering, polylactic acid scaffolds have been considered the most promising ones due to their good biocompatibility, non-toxic biodegradability and bioresorbability. In this work, we evaluated the physiological response of human foetal osteoblast cells, in terms of cell proliferation and osteogenic differentiation, within oxygen plasma treated 3D-printed polylactic acid scaffolds, obtained by fused deposition modelling. The obtained data suggested that 3D-printed polylactic acid scaffolds represent promising biomaterials for medical implantable devices in the orthopaedic field and have the potential to increase patients’ quality of life.

**Abstract:**

Large bone defect treatments have always been one of the important challenges in clinical practice and created a huge demand for more efficacious regenerative approaches. The bone tissue engineering (BTE) approach offered a new alternative to conventional bone grafts, addressing all clinical needs. Over the past years, BTE research is focused on the study and realisation of new biomaterials, including 3D-printed supports to improve mechanical, structural and biological properties. Among these, polylactic acid (PLA) scaffolds have been considered the most promising biomaterials due to their good biocompatibility, non-toxic biodegradability and bioresorbability. In this work, we evaluated the physiological response of human foetal osteoblast cells (hFOB), in terms of cell proliferation and osteogenic differentiation, within oxygen plasma treated 3D-printed PLA scaffolds, obtained by fused deposition modelling (FDM). A mechanical simulation to predict their behaviour to traction, flexural or torque solicitations was performed. We found that: 1. hFOB cells adhere and grow on scaffold surfaces; 2. hFOB grown on oxygen plasma treated PLA scaffolds (PLA_PT) show an improvement of cell adhesion and proliferation, compared to not-plasma treated scaffolds (PLA_NT); 3. Over time, hFOB penetrate along strands, differentiate, and form a fibrous matrix, tissue-like; 4. 3D-printed PLA scaffolds have good mechanical behaviour in each analysed configuration. These findings suggest that 3D-printed PLA scaffolds could represent promising biomaterials for medical implantable devices in the orthopaedic field.

## 1. Introduction

Annually, numerous bone grafts are required for the treatment of severe bone fracture, secondary to bone loss from trauma, congenital anomalies, illnesses including cancer, and aging [1]. To date, autografts, allografts, and xenografts represent the conventional treatments for bone defects, even if several limitations, such as pain, donor site morbidity, rejection, transmission of diseases, and high cost restrict their use [2,3]. The tissue engineering approach, in the last years, has offered a new alternative to conventional bone grafts, developing systems able to address clinical needs and to overcome these limitations [4]. The bone tissue engineering (BTE) approach harnesses biomimetic materials, stem cells and growth factors, aiming to develop scaffolds able to mimic the native bone tissue and improve osteoregenerative properties allowing or enhancing host integration, adhesion, cell viability, proliferation, differentiation, and vascularisation, thereby limiting the disadvantages of traditional grafts [5,6]. Therefore, the design and development of bone functional substitutes plays an increasingly important role in the use of biomedical devices for the repair of damaged tissues [7]. Biomimetic substitutes must provide a provisional matrix that offers a specific environment and architecture to bone cells for three-dimensional (3D) tissue formation [8]. Furthermore, ideal scaffolds for BTE applications must possess several biological (biocompatibility, biodegradability, bio functionality and bioactivity), structural (porosity, pore size, pore interconnection), mechanical (tensile strength, compressive strength, elongation at break) and surface (roughness, composition, surface energy or tension) requirements [9,10]. So, over the past years, BTE research has focused on the study and realisation of new biomaterials with desiderata features, by using automatic, rapid, precise, and economical approaches [11]. Biomaterials currently used for scaffold manufacturing include: (i) bioceramics, such as hydroxyapatite or calcium phosphates, exhibiting good osteointegration, osteoconductivity and compressive strength [12,13,14]; (ii) natural polymers including collagens, hyaluronic acid, and fibrin, showing high osteoconductivity and biocompatibility [15,16]; (iii) synthetic polymers, such as polyethylene glycol (PEG), polycaprolactone (PCL), and polylactic acid (PLA), displaying good biocompatibility and mechanical strength [17,18]; and (iv) hydrogels, hydrated polymer chains, supporting cell adhesion and migration, and facilitating growth factors’ incorporation and targeted release [19]. Among these biomaterials, synthetic polymers received particular attention in BTE applications due to their controlled physicochemical and mechanical properties, degradation rate and processability compared to other materials such as metals and ceramics [20]. Additionally, biodegradable materials are considered the best candidates as scaffolds for tissue regeneration, allowing the regeneration and substitution of the damaged area with newly formed tissue. In this context, bioabsorbable polymers gained great attention for the development of three-dimensional scaffolds, due to their ability to provide support for cells to repair the host tissue and degradability by simple hydrolysis. Notably, the hydrolysis of these three-dimensional scaffolds leads to the formation of products that can be easily metabolised by the human body, i.e., lactic acid [21,22]. Further, synthetic materials can produce scaffolds with definite pore size and interconnections, morphological structure, and anisotropies, by specific manufacturing techniques, improving cell adhesion, migration, proliferation, and differentiation [23].

Aliphatic polyesters, such as polylactic acid (PLA), represent the most common and most promising polymers, due to their biocompatibility, non-toxic biodegradability and bioresorbability in the production of sutures, manufactured orthopaedic devices and support for tissue regeneration [24,25]. Although PLA scaffolds can be produced by different bio-fabrication approaches, such as injection moulding, extrusion, film casting and electrospinning, fused deposition modelling (FDM) is the most interesting technique. FDM is an easy printing technology that uses polymeric filaments to create 3D geometries based on sequential deposition of layers of directionally aligned microfilaments. This technology allows for obtaining scaffolds with appropriate interconnectivity and controlled porosity for effective cell adhesion and vascularisation, resulting in tissue ingrowth [26,27,28].

In addition, with the aim of improving this desired outcome, scaffolds can be suitably plasma treated to enhance surface properties and further promote a positive biological response. In this respect, cold atmospheric plasma, carried out in the presence of helium, was previously assessed as a modification tool for PLA scaffolds to evaluate osteoblast and mesenchymal stem cell attachment [29]. The effects of the treatment have been also considered to modify PLA-Ti6Al4V composites with oxygen or air, the latter providing better mechanical and bioactivity properties [30].

In this work, the potential of plasma treatment was implemented, exposing 3D-printed PLA scaffolds, obtained by FDM, to oxygen, with the aim of clearly assessing the role of the induced modifications on a simple and repetitive pattern, not resembling the physiological architecture of bone tissue. The physiological response of human foetal osteoblast cells (hFOB) was then evaluated in terms of cell proliferation and osteogenic differentiation. PLA scaffolds were also analysed by means of mechanical simulations to evaluate their behaviour to traction, flexural or torque solicitations, in order to assess the potential capability for future implantable applications.

## 2. Materials and Methods

### 2.1. 3D Printing Method and Scaffold Characterisation

Testing scaffolds were designed by stacking polymeric layers in the −60°/0°/60° directions (sample size 12 × 12 × 2 mm^3^). The geometric model was imported in ideaMaker (Raise 3D Inc., Irvine, CA, USA) to prepare the GCode file to drive the N2 FFF 3D printer (Raise 3D Inc., Irvine, CA, USA) and process a polylactic acid (PLA) filament (Formfutura BV, Nijmegen, The Netherlands) for the fabrication stage. The nozzle temperature (0.4 mm diameter) was set at 205 °C while the build platform was set at 40 °C. The morphological assessment of the 3D-printed PLA scaffolds was carried out by means of optical microscopy (Nikon Eclipse 80i) to measure and verify the geometrical consistency of the collected layers with the CAD design.

### 2.2. Scaffold Mechanical Properties Simulation

**Figure 1:**
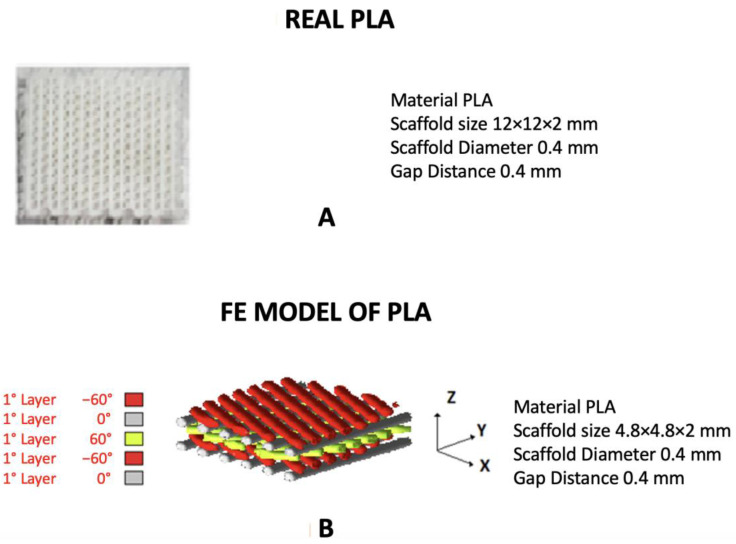
Representation of the 3D-printed scaffold with geometric characteristics (**A**), and the virtual model with layers orientation (**B**).

The mechanical properties of the PLA scaffolds were developed by two different finite element (FE) models and are reported in Table 1. The FE specimen PLA was modelled in a reduced scale 4.8 × 4.8 × 2 mm, overlapping differently oriented rows of filament, according to the real fabricated pattern (Figure 1). The first layer was rotated at −60° with respect to the Y axis while the second one was oriented according to the Y axis. The third and fourth layers were oriented, respectively, at +60° and −60° with respect to the Y axis, while the last layer was oriented according to the Y axis. The model was discretised with 186,798 10-nodes tetrahedral elements and 52,156 nodes. The contact area between a row and the next one, belonging to the next layer, was estimated at about 0.07 mm^2^.

Material properties were imposing by using data from Table 1, and nonlinear elastic analyses were carried out simulating mechanical tests to investigate mechanical behaviour of the scaffolds subjected to a traction, flexural or torque solicitations.

**Figure 2:**
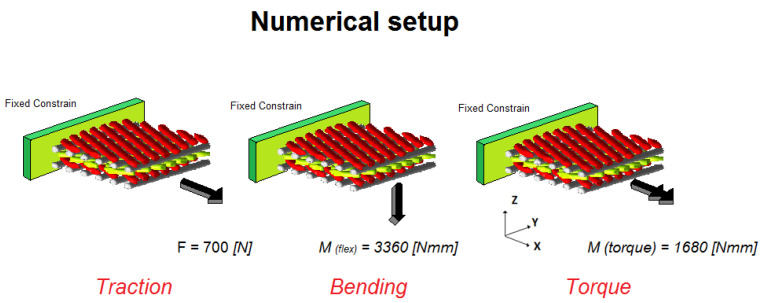
Boundary conditions of numerical setup.

Final loads were calculated by performing benchmarks simulations while continuously increasing the load in a tensile test, since the critical value of stress was reached. The normal load was imposed by fixing 500 nodes at the base of the model and imposing a distributed vertical load of 700 N, as depicted in Figure 2. The bending solicitation was imposed by applying a horizontal distributed load, along the Z-axis, of 3360 Nmm. Finally, a torque moment of 1680 Nmm was imposed to investigate the torsion stiffness, along the X-axis.

### 2.3. Plasma Treatment of 3D-Printed PLA Scaffolds

The oxygen plasma treatment of scaffolds was performed using a RIE plasma etcher SI 591 (SENTECH Instruments GmbH, Berlin, Germany). 3D-printed PLA scaffolds were placed inside the high vacuum reaction chamber where 60 cm^3^/min of O_2_ gas were introduced to expose the scaffolds to a 100 W power, 10 Pa plasma for 10 min. After completing the process, the chamber was ventilated, and the scaffolds were removed and used for the cell adhesion step.

### 2.4. In Vitro Studies

#### 2.4.1. Cell Culture

Human foetal osteoblast cell line hFOB 1.19 were obtained from the American Type Culture Collection (ATCC, Manassas, VA, USA). hFOB 1.19 were cultured in 1:1 mixture of Ham’s F12 Medium—Dulbecco’s Modified Eagle’s Medium (D8437, Merk Life Science S.r.l., Milan, Italy), supplemented with 2.5 mM L-glutamine (G7513, Merk Life Science S.r.l., Milan, Italy), 0.3 mg/mL G418 (4727878001, Merk Life Science S.r.l., Milan, Italy); 10% Foetal Bovine Serum (F7524, FBS, Merk Life Science S.r.l., Milan, Italy) and 1% penicillin/streptomycin/amphotericin (A5955, Merk Life Science S.r.l., Milan, Italy) and incubated in a humidified atmosphere containing 5% CO_2_ at 37 °C. The medium was replaced twice a week and cells were split at about 80% of confluence.

#### 2.4.2. Cells Viability and Proliferation on 3D-Printed PLA Scaffolds

Before performing the in vitro studies, the 3D-printed PLA scaffolds underwent UV sterilization treatment for 2 h to eliminate any possibility of contamination,

Cell viability assay of hFOB cells seeded on 3D-printed PLA scaffolds was performed by MTT [3-(4,5-dimethylthia- zol-2-yl)-2,5-diphenyltetrazolium bromide] assay (M2128, Merk Life Science S.r.l., Milan, Italy). Specifically, 1 × 10^6^ cells were cultured on 3D-printed PLA scaffolds, in a 24-well plate, with a specific medium and incubated in a humidified atmosphere containing 5% CO_2_ at 37 °C for 4 h. Successively, the growth medium was added to completely cover the scaffold and the plates were re-incubated, as above, for 1, 3 and 7 days. After 1, 3 and 7 days of incubation, the medium from each well was removed and replaced with 200 μL of MTT solution (1 mg/mL in FBS-free medium). Following, 2 h incubation at 37 °C and 5% CO_2_, MTT solution was removed, each well was washed two times using cold PBS 0.01 M, and the formed crystals were melted using 200 μL of DMSO (A3672,0250, PanReac AppliChem, ITW Reagents, S.R.L., Monza, Italy). Next, the absorbance at 540 nm was read using a synergy HT plate reader (BioTek Instruments, Inc., VT, USA).

hFOB cell proliferation on 3D-printed PLA scaffolds was determined using DAPI staining as previously reported in [31]. Briefly, for DAPI staining, hFOB-PLA scaffolds were fixed in 4% PFA (J61899, ThermoFisher, Waltham, MA, USA), washed three times in PBS (D8537, Merk Life Science S.r.l., Milan, Italy), permeabilised in 0.3% Triton X-100 (T8787 Merk Life Science S.r.l., Milan, Italy) for 10 min and the nuclei stained with Mounting Medium with DAPI—Aqueous Fluoroshield (AB104139, Abcam, Milan, Italy). The images were acquired using a Leica DMI4000B fluorescence microscope (three digital images/scaffold) and the nuclei counted by Fiji image J recognition software. The proliferation rate differences were assessed using One-way ANOVA test with Holm test as post-hoc for multiple comparisons. At each time point, the biological tests were performed in triplicate.

For cell viability and proliferation assays, the cells cultured on the well with the same seeding density as the scaffolds were used as control (CTRL, only cells).

#### 2.4.3. hFOB Osteogenic Differentiation on 3D-Printed PLA Scaffolds

For osteogenic induction, 1 × 10^6^ hFOB cells were plated on 3D-printed PLA scaffolds in culture medium and incubated in a humidified atmosphere containing 5% CO_2_ at 37 °C. The culture medium was completely replaced twice a week and scaffolds were analysed on day 1, 3 and 7.

#### 2.4.4. Extracellular Matrix Mineralisation on 3D-Printed PLA Scaffolds

The extracellular matrix (ECM) mineralisation of hFOB cells on 3D-printed PLA scaffolds was analysed by Alizarin Red S staining. Alizarin Red S solution (A5533, Merk Life Science S.r.l., Milan, Italy) was prepared according to the manufacturer’s protocol and performed as reported in [32,33]. Briefly, after 1, 3 and 7 days, hFOB-PLA scaffolds were washed with PBS and fixed in 4% PFA for 15 min at room temperature. Then, they were washed three times with H_2_O, 2% Alizarin Red S stain solution was added and the plate with scaffolds was incubated for 30 min at room temperature. Finally, scaffolds were washed five times with H_2_O to remove the staining solution surplus and mounted. The stained scaffolds were acquired by using a Leica DMI 4000B microscope (Leica Microsystems S.r.l., Milano, Italy) and optical density was quantified using Fiji image J software (three sections/scaffold).

#### 2.4.5. Gene Expression Profile of hFOB on 3D-Printed PLA Scaffolds

For gene expression analysis, total RNA from hFOB cells growth on 3D-printed PLA scaffolds was isolated by using Trizol Reagent (15596026, Life Technologies, Carlsbad, CA, USA), according to the manufacturer’s instructions. The concentration and quality of RNA were determined by Nanodrop 1000 Spectrophotometer (ThermoFisher, Waltham, MA, USA). cDNA was synthesised from an equal amount of total RNA using ImProm-II Reverse Transcription System (A3800, Promega, Milan, Italy). Quantitative real-time PCR (qRT-PCR) was performed using a 7500 Fast Real-Time PCR System with Sso Advanced universal SYBR1 Green supermix (2X) (1725271, Bio-Rad Laboratories, Hercules, CA, USA). Specific primers for each of the investigated gene were designed using primer blast and selecting exon-exon junctions on mRNA as the target region for annealing, as already reported [13,34]. Each sample was tested in triplicate and gene expression was assessed using the 2^−ΔΔCt^ method. RNA from hFOB cells without PLA scaffolds (only cells-control) was used as reference for relative quantification. The results were expressed as fold change with respect to control cells. The following primers for real-time PCR were used: Osteocalcin (BGLAP), Alkaline Phosphatase (ALPL), Transforming Growth Factor Beta 1 (TGFb1), Collagen Type 1 (COL1A1) and Collagen Type 2 (COL2A1). Oligonucleotide sequences are reported in Table 2. Results were normalised to the levels of Glyceraldehyde 3-Phosphate Dehydrogenase (GAPDH).

### 2.5. Raman Spectroscopy Analysis of hFOB-PLA Scaffolds

Raman spectra were acquired by using a high-resolution micro-Raman spectrometer (LabRAM HR800 from Horiba Jobin-Yvon) equipped with a Peltier cooled CCD detector (Synapse—Horiba). Laser radiation from a He-Ne (632.8 nm) was focused with a power of 0.9 mW onto the samples by a long working distance 50X objective mounted on an Olympus microscope BX41. The spectroscopic signals were collected in the backscattering configuration with integration times of 30 s.

### 2.6. Statistical Analysis

Data were analysed either as raw data or as mean ± standard error (SE), as appropriate. Differences between several timepoints of hFOB cultured on 3D-printed PLA scaffolds were evaluated by using One and two-way ANOVA with post hoc Holm test, where appropriate. *p* < 0.05 was considered to be significant.

## 3. Results and Discussion

### 3.1. 3D-Printed PLA Scaffolds Characterisation and Mechanical Properties

**Figure 3:**
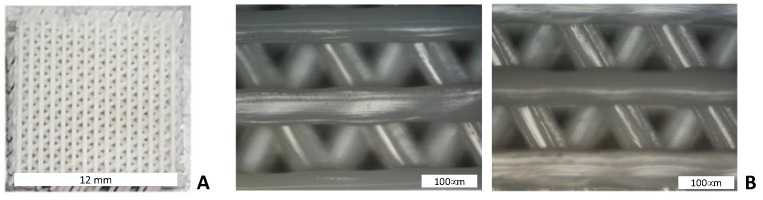
Representative image of a 3D-printed PLA scaffold (**A**) and morphological observations (**B**).

The 3D-printed PLA scaffolds and the related pattern are shown in Figure 3. The collected strands show an average value of 367.27 ± 24.10 µm, slightly differing from the nominal diameter of 400 µm, due to a not homogenous deposition as a “wavy” shape can be observed, possibly induced by an uneven cooling shrinkage. Similarly, a dimensional mismatch in the gap between strands occurred with respect to the designed one, i.e., 400 µm, being 439.61 ± 12.42 µm.

**Figure 4:**
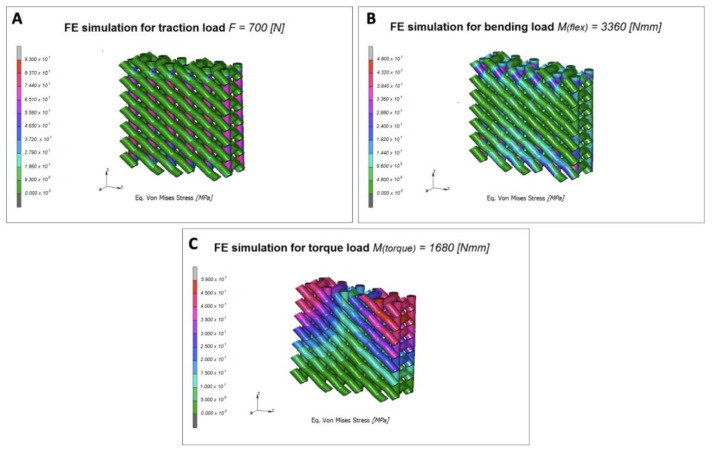
(**A**) Contour maps of eq. Von Mises stress for traction load. (**B**) Contour maps of eq. Von Mises stress for bending load. (**C**) Contour maps of eq. Von Mises stress for torque moment load.

Results of the Finite Element (FE) analysis for tensile load are reported in Figure 4A, depicted as contour maps of Eq. Von Mises stress. The obtained values are approximately 93 MPa for stresses and 0.65 mm for displacements, while related strain has a value of 299 µm/mm. The bending solicitation induces a maximum stress of 48 MPa, as reported in Figure 4B. Related strain has a value of 112 µm/mm, while displacements are about 0.49 mm. Finally, the torsion solicitation produced a maximum equivalent Von Mises stress of 59 MPa (Figure 4C), the related strain has a value of 128 µm/mm, while displacements are 0.53 mm. Thanks to the spatial configuration of the strands, the printed scaffold can react to the imposed solicitation with a much greater number of rows spreading stresses along all of them, thus reducing the final value of the maximum stress detected. These results are in agreement with other literature data in which compression tests were performed to evaluate the mechanical properties of the 3D-printed PLA scaffolds using a universal material testing machine and obtaining values of tensile stress ranging from about 70 MPa and strain of 0.6 mm/mm. Moreover, the value of the tensile strength of PLA scaffold is comparable with the physiological ultimate stress of cortical bone ranging from about 100–150 MPa [35,36]. These data suggest that the 3D-printed PLA scaffold have good mechanical behaviour, in each analysed configuration, and can be used in future applications for medical implantable devices.

### 3.2. Osteoconductive Response of Human Foetal Osteoblast Cells within 3D-Printed PLA Scaffolds

**Figure 5:**
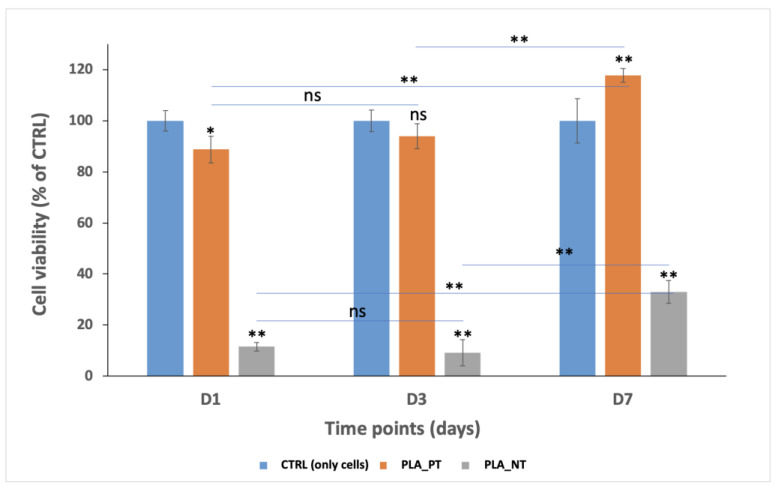
Cell viability (MTT assay) after 1, 3, and 7 days (D1, D3 and D7) of hFOB cells culture on PLA_NT and PLA_PT scaffolds compared to the control (CTRL, only cells). Data are reported as mean ± standard deviation obtained on 3 scaffolds. * *p* < 0.05 and ** *p* < 0.01 show significant differences between the different time points and scaffolds, as reported by the Holm post-hoc test. ns = not significant.

To increase the rate of hFOB cells’ adhesion and distribution along scaffold cavities, we performed O_2_ plasma treatment on 3D-printed PLA scaffolds. Cell viability and proliferation of hFOB cells cultured for 1, 3, and 7 days (D1, D3 and D7) on PLA not treated (PLA_NT) and PLA plasma treated (PLA_PT) scaffold surfaces were assessed. The results are shown in Figure 5.

Cell viability performed by MTT assay showed that the viable cells in PLA_PT and PLA_NT increase over time from D1 to D7, even if the respiratory activity in PLA_NT was lower for both the PLA_PT and control (CTRL, only cells). Specifically, the percentage of viable cells was 88.78 ± 4.84% at D1, 93.93 ± 5.24% at D3, and 117.85 ± 2.75% at D7 for FDM4_PT; while 11.47 ± 1.66% at D1, 9.11 ± 5.21% at D3, and 32.95 ± 4.35% at D7 for PLA_NT compared to 100% of the CTRL group. Further, these data show that the respiratory activity of hFOB cells cultured on PLA_PT scaffolds was 7.7-, 10.3- and 3.57-fold higher than PLA_NT, respectively, from D1 to D7.

These results highlight that the plasma treatment creates available hydroxyl groups on the PLA surface, increasing its wettability and so favouring greater cell adhesion, attachment, and growth, which is consistent with the literature [37,38].

**Figure 6:**
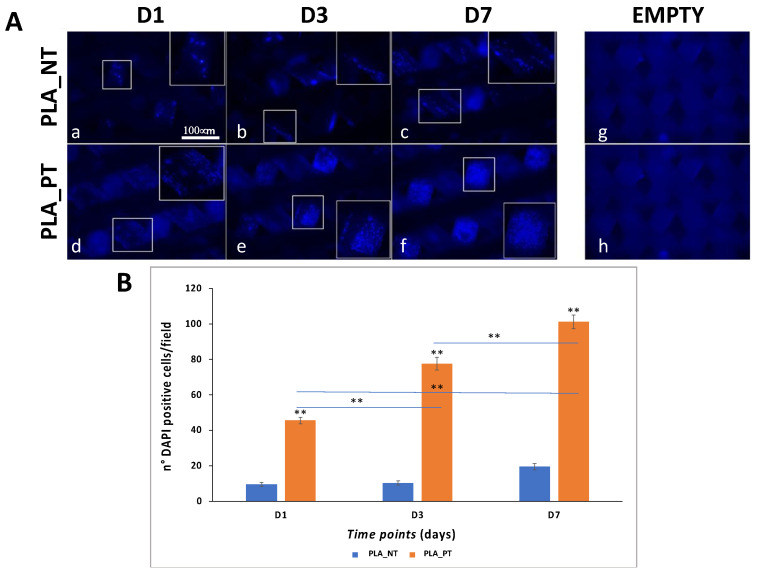
Cell proliferation analysis of hFOB cells cultured on PLA_NT and PLA_PT scaffolds for 1, 3 and 7 days (D1, D3 and D7) by DAPI staining. (**A**) Representative DAPI images of: hFOB cells cultured on PLA_NT (**a**–**c**) and PLA_PT (**d**–**f**) scaffolds, empty scaffolds PLA_NT (**g**), and PLA_PT (**h**). Scale bar 100 mm. (**B**) Graphical representation of the number of DAPI positive cells/field. Data are reported as mean ± standard deviation obtained on 3 scaffolds. ** *p* < 0.01 indicates significant differences between the different time points and scaffolds, as reported by the Holm post-hoc test.

Similar results were obtained by DAPI staining, highlighting that in PLA_NT scaffolds there is a lower number of hFOB cells compared to PLA_PT scaffolds at all analysed time points *(*Figure 6). In addition, we performed a DAPI positive cell count to quantify the cell proliferation rate between the PLA_NT and PLA_PT scaffolds. The results showed that the cell number in PLA_PT was 4.75-fold, 7.46-fold, and 5.16-fold higher than in PLA_NT scaffolds, for D1, D3 and D7, respectively. In addition, the cell number for PLA_PT after day 7 was 2.2-fold and 1.3-fold significantly higher than D1 and D3, respectively.

Data obtained from both MTT and DAPI analyses showed increased viability, adhesion, and proliferation of hFOB cells cultured on PLA_PT scaffolds. These data agree with a previous report showing that 3D-printed PLA scaffolds support the cell adhesion, attachment, growth, and proliferation of osteoblast-like cells [39].

Since cell viability and proliferation analyses showed that the cell number in PLA_NT scaffolds was very low, we decided to analyse the osteogenic differentiation of hFOB only on the PLA_PT scaffolds.

### 3.3. Osteoinductive Response of Human Foetal Osteoblast Cells within 3D-Printed PLA Scaffolds

**Figure 7:**
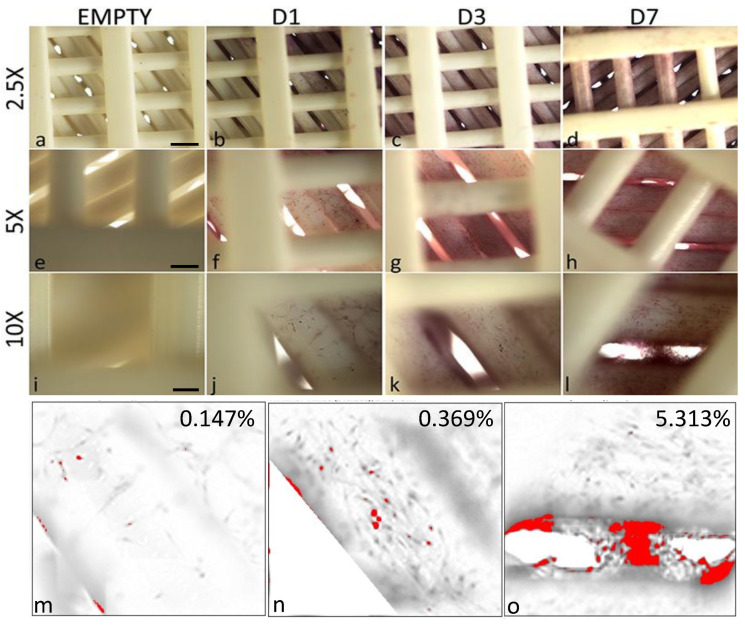
Cell mineralisation analysis of hFOB cells cultured on PLA_PT scaffolds for 1, 3 and 7 days (D1, D3 and D7) by Alizarin Red S staining. Representative images of AR S staining: (**a**,**e**,**i**) empty PLA_PT scaffold; (**b**–**d**,**f**–**h**,**j**–**l**) hFOB cells cultured on PLA_PT scaffolds for D1, D3 and D7, respectively; (**m**–**o**) AR S intensity quantification is reported as % area = percentage of pixels in the image highlighted in red using ImageJ threshold. Scale bar 200 mm (**a**–**d**), 100 mm (**e**–**h**), 50 mm (**i**–**l**).

To evaluate hFOB osteogenic differentiation on PLA_PT scaffolds, we analysed the extracellular matrix mineralisation, in terms of calcium deposits, by Alizarin red S (AR S) staining. AR S staining was performed after 1, 3, and 7 days of hFOB growth on scaffolds. AR S staining representative images are shown in Figure 7, displaying an increase in mineralisation over time from D1 to D7. Further, as it is possible to note in the magnification of Figure 7l, after 7 days of culture on the scaffold, the cells formed a fibrous matrix between the gaps resembling the extracellular matrix of the bone tissue. AR S intensity quantification displayed an increase in the matrix mineralisation from D1 to D7 of about 0.147%, 0.369% and 5.313%, respectively.

**Figure 8:**
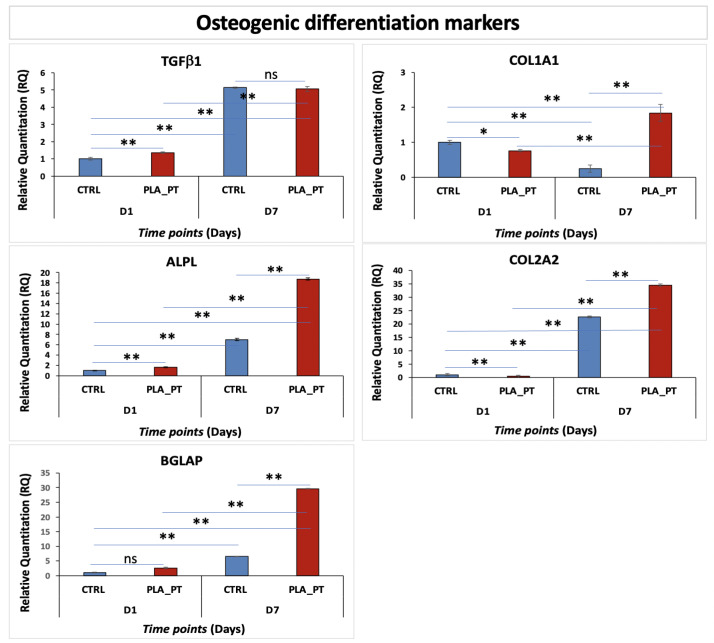
Gene expression profile of hFOB cells cultured on PLA_PT scaffolds for 7 days. Glyceraldehyde-3-phosphate dehydrogenase (GAPDH) has been used as an endogenous control. One-way ANOVA test *p*-value is reported and * indicates significant (*p* < 0.05) differences between groups as reported by the post-hoc test. * *p* < 0.05; ** *p* < 0.01; ns = not significant.

To further evaluate the osteogenic differentiation of hFOB cells cultured on PLA_PT scaffolds, we performed gene expression profiling by quantitative Realtime-PCR (qRT-PCR) at early (D1) and late (D7) time points. Specifically, we evaluated osteogenic differentiation markers functionally related to early osteogenic differentiation (TGFβ1 and ALPL), mineralisation and extracellular matrix maturation (COL1A1, COL2A1, BGLAP) [40,41]. The results are shown in Figure 8.

These data showed that hFOB cells cultured on PLA_PT scaffolds exhibited a TGFβ1 expression level higher than the control (cells cultured in adhesion on the plate, CTRL) only at D1 (1.36 ± 0.03 vs. 1.07 ± 0.07), while at D7, no difference was detectable (5.13 ± 0.1 vs. 5.06 ± 0.02). Instead, at D7, both samples (CTRL and PLA_PT) showed an increase in TGFβ1 expression of about 5-fold compared to D1.

The early osteogenic marker ALPL increased its expression from D1 to D7 on PLA_PT scaffolds by 1.67 ± 0.07 and 18.7 ± 0.22, respectively, compared to the control (1.13 ± 0.06 and 7.01 ± 0.21, for D1 and D7). Finally, expression levels of mineralisation and extracellular matrix maturation markers, COL1A1 and COL2A1, showed a significant increase, only at D7, of about 1.84 ± 0.34 for COL1A1 and 34.5 ± 0.16 for COL2A1. In contrast, BGLAP was overexpressed, starting as early as day 1, by about 2.62 ± 0.21 and 29.6 ± 0.11 at D7, compared to the CTRL (about 1.1 ± 0.10).

The improved expression of TGFβ1 at the early stage of cell growth (D1) and the reduction at D7 is coherent with previous reports, suggesting that this gene is important during the early differentiation of osteoblasts, but it inhibits their late differentiation into osteocytes [42,43]. TGFβ1 plays an important role in the proliferation and early differentiation of osteoblasts improving intracellular Ca^+2^ transport. Further TGFβ1 plays a critical role in bone remodelling, stimulating matrix protein synthesis [44,45]. Further, it has also been demonstrated that ALPL, COL1A1, COL2A1 and BGLAP play a role in cell adhesion, proliferation, extracellular matrix maturation and differentiation of the osteoblast phenotype [46,47]. These findings agreed with our data showing a high level of COL1A1, COL2A1, ALPL and BGLAP in hFOB cultured on a PLA scaffold for 7 days.

### 3.4. Raman Measurement

**Figure 9:**
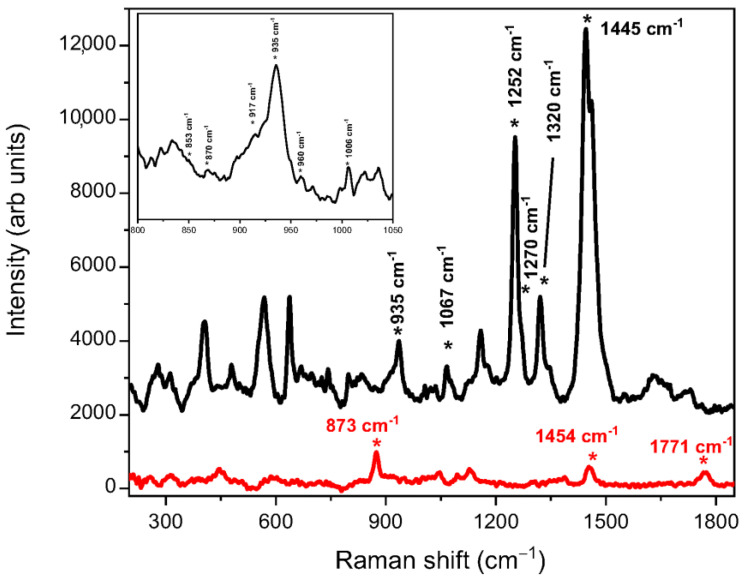
Raman spectra of the pristine PLA scaffold (red line) and of the cell deposited on PLA (black line) and cultured for 7 days. Lorentzian profiles have been adopted to separate the vibrational contributions of the amorphous calcium phosphate at 935 cm^−1^ and of calcium carbonate at 1065–1070 cm^−1^ and fit the experimental data. In the inset, the Raman spectrum of cells deposited on PLA is highlighted in the range between 800 and 1050 cm^−1^. The symbol * indicates the peak position for which the centre-frequency value is indicated.

To detect the simultaneous presence of the mineral and organic composition in bone cultures, cell cultured scaffolds were analysed by Raman spectroscopy. In Figure 9, the Raman spectra of the PLA scaffold, before (red line) and after (black line) 7 days of cell culture (black line), are compared.

The Raman signal coming from the pristine PLA shows the characteristic band at 873 cm^−1^ due to the stretching vibration of the C–COO group. We also observed the bands centred at 1454 cm^−1^ and 1771 cm^−1^ arising from the CH_3_ asymmetric bending and the C=O stretching modes, respectively. After depositing the cells on the PLA scaffold and leaving them in culture for 7 days, we noticed the appearance of a band at 935 cm^−1^. It is attributed to the heterogeneous stretching vibration of P–O–C in the amorphous calcium phosphate, as proof of the incipient mineralisation process. Other deformation modes of phosphate are associated with the peaks at 404 and 568 cm^−1^ [48]. By the ratio between the intensities of the amorphous calcium phosphate band and the one of carbonate at 1065–1070 cm^−1^ (0.65, as obtained by the fitting of the peaks), we accounted for the substitution of carbonate through culture time [49]. We also distinguished the characteristic peaks of the proteinaceous extracellular matrix. In particular, the peaks at 853, 870 and 917 cm^−1^ (highlighted in the inset of Figure 7) are distinctive of the collagen proline and hydroxyproline matrix, and the peak at 1006 cm^−1^ is due to the phenylalanine ring breathing mode [50].

The peak at 1252 cm^−1^ and the shoulder at 1270 cm^−1^ are assigned to the secondary structures of collagen (region of amide III); specifically, they are due to the collagen β-sheet and α-helix, respectively. In the figure, the band at 1320 cm^−1^ due to CH deformation is highlighted, as well as one at 1445 cm^−1^, which is due to CH_2_ wagging. Furthermore, the range between 1580 and 1730 cm^−1^ is typical of the amide I vibrations of the protein.

## 4. Conclusions

In this work, we evaluated the physiological response of human foetal osteoblast cells (hFOB), in terms of cell proliferation and osteogenic differentiation, cultured on O_2_ plasma treated 3D-printed PLA scaffolds. The 3D-printed PLA scaffolds were obtained by fused deposition modelling (FDM) and mechanical simulation to investigate their behaviour when traction, flexural or torque solicitations were performed. Our findings show that hFOB seeded on plasma treated PLA scaffolds are not only able to adhere and grow on their surfaces, but over time, are able to penetrate along strands, differentiate and form a tissue-like fibrous matrix between the gaps. Furthermore, the results of FE simulations showed that the 3D-printed PLA scaffold demonstrates good mechanical behaviour, in each analysed configuration, that can be used in future applications for medical implantable devices. The above reported findings clearly demonstrate that the osteoconductive and osteoinductive properties of the engineered 3D-printed PLA scaffold could represent a promising biomaterial for bone tissue engineering applications.

## Figures and Tables

**Table 1:** Mechanical properties of 3D-printed PLA scaffolds.

Mechanical Properties		
Impact strength	7.5	KJ/m^2^
Tensile strength	110	MPa
Tensile modulus	3310	MPa
Elongation at break	160	%
Flexural strength	55.2	MPa
Flexural modulus	2392	MPa

**Table 2:** qRT-PCR primer sequences.

Target Gene	Forward	Reverse
**BGLAP**	GGCAGCGAGGTAGTGAAGAG	GATGTGGTCAGCCAACTCGT
**ALPL**	GACCCTTGACCCCCACAAT	CGCCTCGTACTGCATGTCCCCT
**COL1A1**	CCGGAAACAGACAAGCAACCCAAA	AAAGGAGCAGAAAGGGCAGCATTG
**COL2A1**	TGGTCTTGGTGGAAACTTTGCTGC	AGGTTCACCAGGTTCACCAGGATT
**TGFb1**	TGGCGATACCTCAGCAACC	CTCGTGGATCCACTTCCAG
**GAPDH**	GCTCTCCAGAACATCATCCCTGCC	GCGTTGTCATACCAGGAAATGAGCTT

## Data Availability

Not applicable.
